# *Escherichia coli mar* and *acrAB* Mutants Display No Tolerance to Simple Alcohols

**DOI:** 10.3390/ijms11041403

**Published:** 2010-03-31

**Authors:** Jonas Ankarloo, Susanne Wikman, Ian A. Nicholls

**Affiliations:** 1 Bioorganic & Biophysical Chemistry Laboratory, School of Natural Sciences, Linnaeus University, SE-391 82 Kalmar, Sweden; E-Mails: jonas.ankarloo@lnu.se (J.A.); susanne.wikman@lnu.se (S.W.); 2 Department of Biochemistry & Organic Chemistry, Uppsala University, Box 576, SE-751 23 Uppsala, Sweden

**Keywords:** solvent tolerance, salicylate, ethanol, 1-propanol, mar regulon, hydrophobicity, solvent

## Abstract

The inducible Mar phenotype of *Escherichia coli* is associated with increased tolerance to multiple hydrophobic antibiotics as well as some highly hydrophobic organic solvents such as cyclohexane, mediated mainly through the AcrAB/TolC efflux system. The influence of water miscible alcohols ethanol and 1-propanol on a Mar constitutive mutant and a *mar* deletion mutant of *E. coli* K-12, as well as the corresponding strains carrying the additional *acrAB* deletion, was investigated. In contrast to hydrophobic solvents, all strains were killed in exponential phase by 1-propanol and ethanol at rates comparable to the parent strain. Thus, the Mar phenotype does not protect *E. coli* from killing by these more polar solvents. Surprisingly, AcrAB does not contribute to an increased alcohol tolerance. In addition, sodium salicylate, at concentrations known to induce the *mar* operon, was unable to increase 1-propanol or ethanol tolerance. Rather, the toxicity of both solvents was increased in the presence of sodium salicylate. Collectively, the results imply that the resilience of *E. coli* to water miscible alcohols, in contrast to more hydrophobic solvents, does not depend upon the AcrAB/TolC efflux system, and suggests a lower limit for substrate molecular size and functionality. Implications for the application of microbiological systems in environments containing high contents of water miscible organic solvents, e.g., phage display screening, are discussed.

## 1. Introduction

Many, but not all, organic solvents are toxic to microorganisms. The toxicity of organic solvents in two-phase water-solvent systems correlates *inversely* with the log *P*_ow_ (the partition coefficient between *n*-octanol and water) of the solvent; in the range between 1 and 5. The ability of solvent molecules to partition into the water phase and into the cell membrane(s) is of great importance for toxicity as highly non-polar solvents (log *P*_ow_ above 5) show no adverse effect on the metabolic activity in several bacterial species [1]. For readily water-miscible organic solvents such as short-chain alcohols (e.g., methanol, ethanol and 1-propanol), toxicity correlates directly with hydrophobicity [2,3].

The cytoplasmic membrane is the main site of action for organic solvents. Using 1,2,3,4-dihydronaphthalene (tetralin), Sikkema and coworkers [4] demonstrated a decrease in membrane potential (Δp), chiefly dependent on a lowered internal pH and transmembrane ΔpH, in cytochrome C oxidase-reconstituted liposomes. This effect was ascribed to an increased H^+^-permeability of the membrane, and was concomitant with growth arrest in similarly treated sensitive bacteria. Furthermore, cyclic hydrocarbons were shown to dissipate both the pH gradient and the electrochemical potential [5]. Ethanol has been shown to affect the proton motive force [6], and to increase leakage of metabolites from cells [7].

Inducible *multi*-antibiotic-, disinfectant- and oxidative stress agent-tolerance regulons have been identified in several bacterial species [8–10]. The *E. coli mar* operon contains a promotor under negative control of the MarR repressor, which is co-transcribed with the *marA* gene [11]. This gene encodes a transcriptional activator affecting the expression of some 60 chromosomal genes [12] including the up-regulation of the AcrAB plasma membrane efflux transporter and TolC outer membrane channel. As a result, structurally unrelated hydrophobic or amhiphilic compounds such as β-lactams, tetracycline, chloramphenicol, fluoroquinolones [13,14] as well as bile salts [15] and some disinfectants [16,17] may be pumped across both membranes. Null mutations in *marR* cause the overexpression of *marA* and constitutive multi-antibiotic tolerance (the Mar phenotype). In addition, the *mar* operon is inducible in wild-type strains, e.g., by low levels of tetracycline or chloramphenicol, but not ampicillin [18]. In the absence of the AcrAB pump, *marR* mutations fail to display the Mar phenotype [19].

Salicylate has been shown to induce the *mar* operon by directly binding to, and inactivating MarR [20,21]. The redundancy in hydrophobic substance-extruding pumps is evident in that salicylate weakly increases antibiotic tolerance (excluding aminoglycosides) in *marA*-deleted *E. coli* [22]. Indeed, salicylate has been shown to relieve *emrRAB* repression by binding to EmrR [23].

The previous isolation of cyclohexane-tolerant *E. coli* K-12 mutants that showed multi-antibiotic tolerance [24] raised the question as to whether there was a common denominator between solvent- and antibiotic tolerance in *E. coli*. Indeed, one such mutant proved to be *marR* [25], suggesting a common stress-dependent resistance mechanism. Clinical isolates of *E. coli* exhibiting fluoroquinolone resistance, have shown an increased proportion of cyclohexane-tolerant strains [26]. Moreover, plasmid-mediated overexpression of *marA* has been shown to increase tolerance to both cyclohexane and hydrophobic antibiotics [25]. The objective of the present study was to establish if Mar activation could also increase tolerance to water-miscible solvents (ethanol, 1-propanol).

## 2. Results and Discussion

### 2.1. Exponential-phase Killing by Ethanol or 1-Propanol Is Unaffected by the *mar* Phenotype

**Figure 1.**
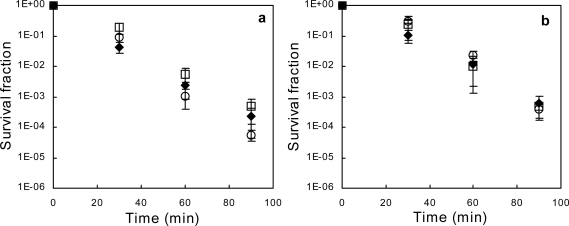
Relative survival of *E. coli* AG100 (black diamonds), AG112 *marR* (open squares) and AG100/Kan Δ(*marCORAB*) (open circles) after exposure to (**a**) 10.0% (w/w) ethanol, or (**b**) 4.05% (w/w) 1-propanol. Cells were cultured in modified LB (see Materials and Methods section) until late exponential phase before the addition of solvent and further incubation. Error bars represent 95% confidence intervals based upon 8 replicates.

At 10.0% (w/w) and 4.05% (w/w), respectively, ethanol and 1-propanol killed the *mar* as well as the parent *E. coli* strains exponentially, with single-hit kinetics (Figure 1). Despite the rapid loss of viability, optical density of the cultures increased slightly during the first 30–60 min after alcohol addition (not shown), which is indicative of a growth in the size of viable bacteria, with no apparent osmotic lysis.

No statistically significant differences between any strains in 30-, 60- or 90-minute survival fractions could be detected, neither in the ethanol-treated (Figure 1a), nor in the 1-propanol-treated (Figure 1b) cultures. The Mar phenotype expressed in *E. coli* AG112 does not protect this strain against ethanol- or 1-propanol-mediated killing, compared to the parent strain. The deletion of *marA* in *E. coli* AG100/Kan does not render this strain more susceptible to ethanol or 1-propanol, thus *mar* induction may not significantly contribute to tolerance towards these alcohols.

Growing *E. coli* are generally more solvent-sensitive than non-growing cells [2], therefore cultures in mid- to late exponential phase were chosen for subsequent solvent addition. In the stationary phase, solvent tolerance mechanisms other than Mar would take effect. Stationary-phase-dependent expression of the alternative sigma factor RpoS up-regulates *uspB*, which is required for stationary-phase resistance to ethanol [30].

Growth is but one indicator of the physiological status of bacterial cells. *E. coli* generation times increase in the presence of low concentrations of ethanol [31], presumably through the diminished cellular energy status. At 5% (v/v), ethanol causes growth arrest after one mass doubling with incomplete septation, also indicating an effect on cell wall synthesis [31]. This growth arrest could be alleviated by dilution with fresh medium. In contrast, the ethanol- and 1-propanol effect observed here is irreversible, killing the cells. In minimal medium, 5% (v/v) of ethanol has been shown to cause cellular lysis in the absence, but not in the presence, of 0.1 M NaCl. Peptidoglycan cross-linking was also shown to decrease [2]. It was proposed that peptidoglycan cross-linking enzymes may be inhibited because ethanol generally weakens hydrophobic interactions within the periplasm as well as the membrane interior, and that this may be overcome by NaCl, as well as other antichaotropic salts. Similar lysis has been reported in *E. coli* grown in Luria-Bertani broth containing up to 5% (v/v) of ethanol [32]. That no gross lysis ensued in our case may be surprising, especially since NaCl was omitted from the growth medium. However, actively growing cells, simultaneously synthesizing poorly cross-linked peptidoglycan and displaying autolysin activity, would be expected to readily lyse. Rapid cellular inactivation due to other mechanisms, at the relatively high alcohol concentrations used here may hence mask any impairment of cell wall synthesis, or autolysins may simply be just as rapidly inhibited.

### 2.2. Salicylate Increases the Killing Rate of Ethanol and 1-Propanol, Irrespective of *mar* Induction

**Figure 2.**
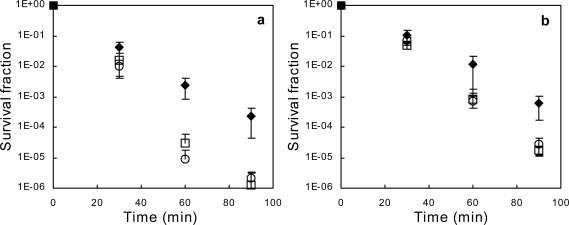
Relative survival of *E. coli* AG100 (open squares) and AG100/Kan Δ(*marCORAB*) (open circles) grown in the presence of sodium salicylate, and *E. coli* AG100 grown in LB without salicylate (black diamonds) after subsequent exposure to (**a**) 10.0% (w/w) ethanol, or (**b**) 4.05% (w/w) 1-propanol. Cells were cultured in modified LB (see Materials and Methods section) containing 2 mM sodium salicylate until late exponential phase before the addition of solvent and further incubation. Error bars represent 95% confidence intervals based upon 8 replicates.

*E. coli* AG100/Kan is a *mar* deletion mutant lacking most of the *mar* operon including the *mar* repressor and Mar activator genes. Salicylate can elicit no MarA-dependent solvent tolerance in this strain, but hypothetically so in the parent strain AG100 as cyclohexane tolerance was earlier shown to increase by salicylate addition (or *marA* overexpression) in *E. coli* W3110; not carrying any *mar* mutation [25]. Addition of 10.0% ethanol to *E. coli* AG100 and AG100/Kan grown in the presence of 2 mM sodium salicylate results in an increased killing rate, compared to AG100 grown without salicylate (Figure 2a). Similarly, salicylate increases the killing rate of 4.05% 1-propanol in both strains (Figure 2b). The 60- and 90-minute survival fractions of salicylate/solvent-exposed AG100 as well as AG100/Kan differ significantly from non-salicylate treated AG100, for both solvents. In contrast, no statistically significant differences between AG100 and AG100/Kan survival rates in the presence of salicylate can be shown. Therefore, salicylate (2 mM) significantly enhances ethanol- and 1-propanol mediated killing of *E. coli* K-12, irrespective of any salicylate-mediated Mar induction. This further strenghtens our view that the Mar phenotype cannot protect *E. coli* against lower primary alcohol (ethanol, 1-propanol) mediated killing.

Interestingly, synergism between a weak acid and ethanol in killing of *E. coli* has been described before [33]. 50 mM of lactate in combination with 5% (v/v) ethanol enhanced killing of *E. coli* O157:H7 about four log units in the exponential phase (first hour after addition), compared to ethanol only. It was also found that both lactate and ethanol independently lowered the cytoplasmic pH of the cells. We have not investigated cellular pH, but salicylate at 2.5 mM, close to the concentration used here, has been shown to increase bacterial sensitivity towards aminoglycosides [34,35]. This has been proposed to partly result from intracellular deprotonation following salicylic acid diffusion across the cytoplasmic membrane [36,37].

### 2.3. Deletion of *acrAB* Does Not Increase Sensitivity to Ethanol or 1-Propanol

**Figure 3.**
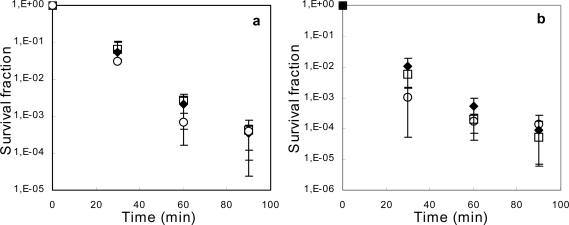
Relative survival of *E. coli* AG100 (black diamonds), AG100A Δ(*acrAB*) (open squares) and AG102K *marR* Δ(*acrAB*) (open circles) after exposure to **a**) 10.0% (w/w) ethanol, or **b**) 4.05% (w/w) 1-propanol. Cells were cultured in modified LB (see Materials and Methods section) until late exponential phase before the addition of solvent and further incubation. Error bars represent 95% confidence intervals based upon 8 replicates.

Ma and coworkers [14] have described the induction of *acrAB* transcription by 4% ethanol, even in a Δ*mar* strain; thus independently of the Mar response. In this study they did establish whether ethanol was a substrate of the AcrAB pump. AcrAB and TolC are both necessary to extrude solvents with a *P*_ow_ greater than 3.4 [38]. We measured the killing rate of ethanol and 1-propanol in Δ(*acrAB*) as well as Δ(*acrAB*) *marR* strains (Figure 3). Again, no statistically significant differences between AG100, AG100A and AG102/K survival rates after ethanol or 1-propanol exposure could be shown. This suggests that the AcrAB efflux pump cannot pump ethanol or 1-propanol across the membrane.

In summary, the fact that salicylate similarly enhanced ethanol- and 1-propanol-mediated killing, and that a lower concentration of the more hydrophobic 1-propanol was required to reach a comparable killing rate, suggest a common, synergistic relationship between salicylate and the alcohols, as well as a common, general killing mechanism presumably based on the partitioning of solvent into the plasma membrane. Neither the Mar phenotype, a well-characterised multi-drug and organic solvent tolerance phenotype, nor any stress-dependent induction of Mar or its central component AcrAB could alleviate the effect of ethanol- and 1-propanol. Collectively, these results establish a lower limit in terms of size and functionality for substrates for the AcrAB pump, information which should prove valuable for understanding resistance mechanisms, and for the use of microorganisms in biotechnological processes involving organic solvents [39,40].

## 3. Experimental Section

### 3.1. Bacterial Strains and Media

*E. coli* AG100 (K-12 *argE3 thi-1 rpsL xyl mtl* Δ(*gal-uvrB*) *supE44*) [27], AG112 (*marR* 5-bp deletion) [28], AG100A (Δ(*acrAB*)::Kan) [19], AG102K (*marR* Δ(*acrAB*)::Kan) [19] and AG100/Kan (Δ(*marCORAB*)::Kan) [29] were all kindly donated by Dr. S. B. Levy, Center for Adaptation Genetics and Drug Resistance, Tufts University School of Medicine, Boston MA, USA. All strains were maintained on Luria-Bertani (LB) Agar (10 g Tryptone (Merck), 5 g Yeast extract (Merck), 5 g NaCl, 12 g agar (Difco) and 10 mL 10% (w/v) d-glucose, separately autoclaved l^−1^); and where appropriate supplemented with 12.5 μg kanamycin mL^−1^.

### 3.2. Solvent Killing Assay

Overnight cultures of *E. coli* in modified LB Broth (10 g Tryptone (Merck), 5 g Yeast extract (Merck) and 10 mL 10% (w/v) d-glucose, separately autoclaved l^−1^) were re-inoculated (1:100) into the same medium with or without sodium salicylate (2 mM) and shaken vigorously at 37 °C. In mid- to late exponential phase (≈5 × 10^8^ cfu mL^−1^; 3–4 h), 1-propanol (4.05%, w/w) or ethanol (10.0%, w/w) was added. Shaking was continued at 37 °C with samples being removed every 30 min for subsequent dilution in cold Dil (1 g Nutrient Broth (Merck), 5 g NaCl L^−1^) before plating on LB Agar. Plates were incubated overnight at 37 °C for the determination of viable counts. Survival at each time point was calculated as fractions of the viable count at the time of solvent addition. Data were statistically treated using the unpaired t-test. Optical density of liquid cultures was measured at 635 nm using an Aquanal-plus Spectro™ water analyser (Riedel-de-Haën, Germany).

## 4. Conclusions

In this study we have investigated the influence of small water miscible alcohols ethanol and 1-propanol on a Mar constitutive mutant and a *mar* deletion mutant of *E. coli* K-12, as well as the corresponding strains carrying the additional *acrAB* deletion. In contrast to hydrophobic solvents, all strains were killed in exponential phase by 1-propanol and ethanol at rates comparable to the parent strain. Although the inducible Mar phenotype of *Escherichia coli* is associated with increased tolerance to multiple hydrophobic antibiotics as well as some highly hydrophobic organic solvents such as cyclohexane, mediated mainly through the AcrAB/TolC efflux system, the Mar phenotype does not appear to protect *E. coli* from killing by these more polar solvents. Interestingly, sodium salicylate, at concentrations known to induce the *mar* operon, was unable to increase 1-propanol or ethanol tolerance. Rather, the toxicity of both solvents was increased in the presence of sodium salicylate. Collectively, the results imply that the resilience of *E. coli* to water miscible alcohols, in contrast to more hydrophobic solvents, does not depend upon the AcrAB/TolC efflux system. Importantly, these results help define the structural limits, in terms of molecular size and functionality, necessary for AcrAB/TolC efflux system substrates.

Furthermore, the inherent stability of microbiological systems, both phage [41] and host cells, towards media containing high contents of organic solvents is of importance when using phage display strategies for screening for recognition motifs selective for relatively hydrophobic targets of nonbiological origin [42]. Moreover, the establishment of phage display protocols suitable for use in media of low dielectricity [43], requires host cell systems capable of withstanding solvent-induced stress. Again, the results presented should assist in the development of such protocols.
